# Prostate Cancer Incidence and Survival, by Stage and Race/Ethnicity — United States, 2001–2017

**DOI:** 10.15585/mmwr.mm6941a1

**Published:** 2020-10-16

**Authors:** David A. Siegel, Mary Elizabeth O’Neil, Thomas B. Richards, Nicole F. Dowling, Hannah K. Weir

**Affiliations:** 1 Division of Cancer Prevention and Control, National Center for Chronic Disease Prevention and Health Promotion, CDC.

Among U.S. men, prostate cancer is the second leading cause of cancer-related death (*1*). Past studies documented decreasing incidence of prostate cancer overall since 2000 but increasing incidence of distant stage prostate cancer (i.e., signifying spread to parts of the body remote from the primary tumor) starting in 2010 (*2*,*3*). Past studies described disparities in prostate cancer survival by stage, age, and race/ethnicity using data covering ≤80% of the U.S. population (*4*,*5*). To provide recent data on incidence and survival of prostate cancer in the United States, CDC analyzed data from population-based cancer registries that contribute to U.S. Cancer Statistics (USCS).* Among 3.1 million new cases of prostate cancer recorded during 2003–2017, localized, regional, distant, and unknown stage prostate cancer accounted for 77%, 11%, 5%, and 7% of cases, respectively, but the incidence of distant stage prostate cancer significantly increased during 2010–2017. During 2001–2016, 10-year relative survival for localized stage prostate cancer was 100%. Overall, 5-year survival for distant stage prostate cancer improved from 28.7% during 2001–2005 to 32.3% during 2011–2016; for the period 2001–2016, 5-year survival was highest among Asian/Pacific Islanders (API) (42.0%), followed by Hispanics (37.2%), American Indian/Alaska Natives (AI/AN) (32.2%), Black men (31.6%), and White men (29.1%). Understanding incidence and survival differences by stage, race/ethnicity, and age can guide public health planning related to screening, treatment, and survivor care. Future research into differences by stage, race/ethnicity, and age could inform interventions aimed at improving disparities in outcomes.

Cases included males with malignant^†^ prostate cancer^§^ and excluded cases diagnosed by autopsy and death certificate only. Incidence data were from USCS during the period 2003–2017 and covered 100% of the U.S. population. Age-adjusted rates were expressed per 100,000 men.^¶^ Trends in incidence were described using annual percent change (APC) and average annual percent change (AAPC) calculated by joinpoint regression. Statistically significant APC and AAPC were different from zero (p<0.05).** Survival data were from CDC’s National Program of Cancer Registries (NPCR)–funded registries that conducted active case follow-up or linkage with CDC’s National Death Index, and covered 94% of the U.S. population.^††^ Survival analysis included cases diagnosed during 2001–2016 with follow-up through December 31, 2016. Relative survival (cancer survival in the absence of other causes of death) was calculated**^§§^** for 1, 5, and 10 years after diagnosis, using expected life tables stratified by age, sex, race/ethnicity, socioeconomic status, geographic location, and calendar year of diagnosis.^¶¶^ Differences between relative survival estimates were determined by comparing 95% confidence intervals (CIs), which allowed for an informal, conservative comparison of estimates. Differences in relative survival were noted when CIs did not overlap.

Incidence and survival were stratified by stage, age, year of diagnosis, and race/ethnicity. There were four categories for race (Black, White, AI/AN, and API) and one for ethnicity (Hispanic). Men categorized by race were all non-Hispanic. Men categorized as Hispanic might be of any race. Stage was defined using Summary Stage, the staging system used by the cancer surveillance community and defined with the following categories: localized (tumor is confined to the organ of origin without extension beyond the primary organ), regional (direct extension of the tumor to adjacent organs or structures or spread to regional lymph nodes), distant (cancer has spread to parts of the body remote from the primary tumor), and unknown.***

During 2003–2017, a total of 3,087,800 new cases of prostate cancer were diagnosed in the United States (Table 1). Over this 15-year period, age-adjusted incidence decreased from 155 per 100,000 in 2003 to 105 in 2017 (Supplementary Table 1, https://stacks.cdc.gov/view/cdc/94592). During 2003–2017, incidence was highest for men aged 70–74 years (764) and Black men (202). Localized, regional, distant, and unknown stage prostate cancer accounted for 77%, 11%, 5%, and 7% of total cases, respectively. The percentage of localized cases decreased from 78% in 2003 to 70% in 2017, and distant cases increased from 4% in 2003 to 8% in 2017. White men had lower percentages of distant (5%) and unknown stage (6%) prostate cancer than did any other race/ethnicity. The overall incidence of prostate cancer decreased during 2003–2017 (AAPC = −2.5%) but increased for cases diagnosed at distant stage (AAPC = 2.2%). More specifically, the increase was observed during 2010–2017 (APC = 5.1%) and began in 2011 or earlier, regardless of race/ethnicity.

During 2001–2016, among 3,104,380 men with survival data, 5-year and 10-year relative survival was 97.6% and 97.2%, respectively (Table 2). Men aged ≤49 years and ≥80 years had the lowest 10-year relative survival (95.6% and 82.7%, respectively). For localized prostate cancer, 10-year relative survival was 100%. Ten-year relative survival for regional, distant, and unknown stage was 96.1%, 18.5%, and 78.1%, respectively. For distant stage prostate cancer, 10-year relative survival was highest for ages 60–64 years (21.8%) and was <20% for ages <55 and ≥70 years.

Comparing 2001–2005 with 2011–2016, 5-year relative survival improved from 97.5% to 99.3% for regional stage and from 28.7% to 32.3% for distant stage prostate cancer (Table 3). During 2001–2016, 5-year survival for distant stage prostate cancer was highest among API (42.0%), followed by Hispanics (37.2%), AI/AN (32.2%), Black men (31.6%), and White men (29.1%). Survival by race/ethnicity showed differences by age (Supplementary Table 2, https://stacks.cdc.gov/view/cdc/94593). For unknown stage prostate cancer, 5-year survival was higher for Hispanic (84.4%) and White men (82.8%) than Black men (79.1%).

## Discussion

Although approximately three fourths of U.S. men with prostate cancer have localized stage at diagnosis, an increasing number and percentage of men have received diagnoses of distant stage prostate cancer. Survival with distant stage prostate cancer has improved, but fewer than one third of men survive 5 years after diagnosis. Survival disparities by age and race/ethnicity were noted for distant stage prostate cancer during all three periods (i.e., 2001–2005, 2006–2010, and 2011–2016) studied.

The U.S. Preventive Services Task Force (USPSTF) has issued several recommendations that discuss the possible benefits and harms of screening for prostate cancer using prostate-specific antigen (PSA).^†††^ In 2012, USPSTF concluded that the benefits of PSA-based screening do not outweigh the harms and recommended against PSA-based screening for prostate cancer for men of all ages. This recommendation likely contributed to a decrease in overall reported prostate cancer incidence and might have contributed to an increase in the percentage and incidence of distant stage prostate cancer (*2*,*3*). Despite decreasing incidence of localized stage prostate cancer, 130,658 to 190,570 new cases were diagnosed each year in the United States during 2003–2017. Even though 10-year survival for localized stage prostate cancer is 100%, many of these patients need treatment, including surgery or radiation, often face long-term effects of their treatment (e.g., urinary incontinence and erectile dysfunction), and ≤6% progress to metastatic prostate cancer (*6*). Improvements in survival for distant stage prostate cancer might reflect changes in clinical management, which includes increased use of new agents and treatment innovations, such as new hormone and antibody therapies (*6*). Despite these improvements in survival, increases in distant stage prostate cancer incidence might have contributed to the plateauing of previously declining prostate cancer mortality during 2013–2017 (*1*,*2*).

Five-year survival for all stages combined was higher for White men than Black or Hispanic men. However, survival for distant stage prostate cancer was higher for Black than White men, which is different from a past study reporting higher survival for White men than Black men during 2001–2009, but with overlapping 95% CIs (*4*). In addition, unknown stage prostate cancer represented a higher percentage of total cases (7%) than distant stage prostate cancer (5%), and survival for unknown stage prostate cancer was higher for Hispanic and White men than Black men. Men in the unknown stage category, who had a 5-year relative survival of 84.3%, might include a mixture of situations, such as men not healthy enough for a staging workup, situations where staging is not needed to guide treatment decisions, lack of access to care, or incomplete recording in the medical record (*7*). Past data suggest that social inequities by race contribute to worse outcomes for Black men than White men with prostate cancer (*8*). Survival based on distant stage and race/ethnicity might need to be interpreted in the context of the incidence and survival for other prostate cancer stages, as well as diagnostic procedures and social determinants of health such as access to care (*7*,*8*).

Although survival by age varied by stage, survival was lowest for ages >75 years for regional, distant, and unknown stage prostate cancer. Lower survival for distant stage at age >75 years compared with younger ages might be secondary to more rapid development of resistant prostate cancer, reduced ability to receive available therapies, and impact of comorbidities (*5*). Ten-year survival was lower for men aged ≤49 years compared with all ages except ≥80 years. Prostate cancer incidence in men ≤49 years has risen over the past 3 decades, and lower survival for this age group has been reported (*9*). Prostate cancer behavior, genetics, family history, and treatment patterns might affect prostate cancer incidence and survival patterns for men aged ≤49 years (*9*).

The findings in this report are subject to least three limitations. First, prostate cancer cases missing from the dataset could result in an undercount of prostate cancer incidence,**^§§§^** and delays in reporting could undercount incidence over the most recent years of the study (*10*). Second, Collaborative Cancer Staging coding, which was used from 2003 to 2015 to code stage data, might explain the lower numbers of unknown stage cases during those years.^¶¶¶^ Finally, confidence intervals could not be generated for all survival results that are rounded to 100.0%, and values listed as 100.0% only mean that no excess deaths were observed.

In 2018, USPSTF issued a new recommendation stating that prostate cancer screening for men aged 55–69 years should be an individualized decision based on personal preferences when weighing the benefits and harms of screening,**** and several professional organizations have similarly recommended shared decision-making for men deciding about prostate cancer screening.^††††^ Understanding incidence and long-term survival by stage, race/ethnicity, and age could inform messaging related to the possible benefits and harms of prostate cancer screening and could guide public health planning related to treatment and survivor care. Further research is needed to examine how social determinants of health affect prostate cancer diagnosis and treatment; findings should inform interventions to decrease disparities in outcomes.

**Summary**

**What is already known about this topic?**

Among U.S. men, prostate cancer is the second leading cause of cancer-related death. The incidence of distant stage prostate cancer (signifying spread to parts of the body remote from the primary tumor) has increased since 2010.

**What is added by this report?**

Additional years of data show continued increases in the incidence of distant stage prostate cancer in the United States. The percentage of distant stage prostate cancer increased from 4% in 2003 to 8% in 2017. Five-year survival for distant stage prostate cancer improved from 28.7% during 2001–2005 to 32.3% during 2011–2016; for the period 2001–2016, 5-year survival was highest among Asian/Pacific Islanders (42.0%), followed by Hispanics (37.2%), American Indian/Alaska Natives (32.2%), Black men (31.6%), and White men (29.1%).

**What are the implications for public health?**

Understanding the disease trends of distant stage prostate cancer and disparities in prostate cancer survival by stage, race/ethnicity, and age can guide public health planning related to screening, treatment, and survivor care.

**TABLE 1:** Age-adjusted incidence* of prostate cancer^†^ and annual percent change (APC) and average APC (AAPC) in rates per 100,000 men, by selected characteristics — U.S. Cancer Statistics, United States, 2003–2017

Characteristic	No., % of total, and rate		AAPC 2003–2017^§^	APC^§^					
	No. (%)^¶^	Rate (95%CI)	AAPC (95% CI)	Yrs	APC1 (95% CI)	Yrs	APC2 (95% CI)	Yrs	APC3 (95% CI)
**Overall**	**3,087,800 (100)**	**128.4 (128.2** to **128.5)**	**−2.5 (−4.1 to **−0.9)**	**2003–2007**	**2.0 (−1.6** to **5.7)**	**2007–2014**	**-6.6 (−8.8** to −4.4)**	**2014–2017**	**1.6 (−4.0** to **7.6)**
**Age group (yrs)**									
≤49	81,420 (3)	5.2 (5.1 to 5.2)	−2.9 (−4.0 to −1.7)**	2003–2009	4.4 (2.2 to 6.6)**	2009–2017	−8.0 (−9.5 to −6.4)**	—^††^	—
50–54	212,288 (7)	134.5 (133.9 to 135.0)	−1.6 (−3.7 to 0.6)	2003–2009	2.7 (0.2 to 5.2)**	2009–2014	−7.4 (−11.7 to −2.8)**	2014–2017	−0.1 (−7.8 to 8.3)
55–59	410,683 (13)	288.0 (287.1 to 288.9)	−1.8 (−3.6 to 0.0)	2003–2008	2.3 (−0.7 to 5.4)	2008–2014	−6.4 (−9.2 to −3.6)**	2014–2017	1.1 (−5.7 to 8.3)
60–64	569,259 (18)	484.7 (483.4 to 485.9)	−1.9 (−3.7 to −0.1)**	2003–2008	1.9 (−1.1 to 5.0)	2008–2014	−6.9 (−9.6 to −4.1)**	2014–2017	2.2 (−4.4 to 9.2)
65–69	658,449 (21)	720.0 (718.3 to 721.8)	−2.0 (−3.8 to −0.1)**	2003–2008	1.4 (−1.8 to 4.8)	2008–2014	−6.8 (−9.8 to −3.8)**	2014–2017	2.5 (−4.3 to 9.8)
70–74	516,620 (17)	764.0 (762.0 to 766.1)	−2.5 (−4.4 to −0.6)**	2003–2007	2.0 (−2.4 to 6.5)	2007–2014	−7.0 (−9.2 to −4.6)**	2014–2017	2.3 (−4.8 to 9.9)
75–79	346,422 (11)	693.6 (691.3 to 695.9)	−3.1 (−4.9 to −1.3)**	2003–2007	0.6 (−3.1 to 4.5)	2007–2014	−8.0 (−10.2 to −5.9)**	2014–2017	4.1 (−3.1 to 11.7)
≥80	292,659(9)	473.1 (471.4 to 474.8)	−4.6 (−5.9 to −3.2)**	2003–2007	−2.7 (−5.7 to 0.4)	2007–2013	−9.5 (−11.7 to −7.2)**	2013–2017	1.4 (−2.4 to 5.2)
**Race/Ethnicity^§§^**									
White	2,296,805 (74)	122.2 (122.0 to 122.3)	−2.7 (−4.7 to −0.5)**	2003–2007	2.1 (−2.5 to 7.0)	2007–2014	−7.0 (−9.4 to −4.6)**	2014–2017	1.6 (−6.7 to 10.7)
Black	451,822 (15)	202.3 (201.7 to 203.0)	−2.6 (−3.9 to −1.2)**	2003–2009	−0.5 (−2.2 to 1.1)	2009–2014	−6.6 (−9.5 to −3.7)**	2014–2017	0.4 (−4.3 to 5.3)
AI/AN	12,232 (0)	87.9 (86.2 to 89.6)	−3.4 (−5.3 to −1.4)**	2003–2009	−0.6 (−3.1 to 1.8)	2009–2014	−7.9 (−11.8 to −3.8)**	2014–2017	−1.1 (−7.7 to 6.0)
API	62,184 (2)	67.2 (66.6 to 67.7)	−3.6 (−6.5 to −0.6)**	2003–2011	−3.3 (−4.8 to −1.7)**	2011–2014	−11.1 (−23.5 to 3.3)	2014–2017	3.6 (−3.1 to 10.8)
Hispanic	196,506 (6)	106.0 (105.5 to 106.5)	−3.8 (−4.9 to −2.6)**	2003–2008	−0.5 (−2.5 to 1.5)	2008–2014	−7.4 (−9.1 to −5.7)**	2014–2017	−1.5 (−5.7 to 2.9)
**Stage^¶¶^**									
Localized	2,373,517 (77)	98.1 (98.0 to 98.3)	−3.3 (−5.1 to −1.4)**	2003–2007	3.1 (−1.2 to 7.5)	2007–2014	−8.0 (−10.1 to −5.9)**	2014–2017	−0.1 (−7.2 to 7.5)
Regional	344,750 (11)	13.5 (13.4 to 13.5)	0.2 (−1.5 to 2.1)	2003–2007	3.3 (−0.9 to 7.7)	2007–2013	−3.2 (−6.1 to −0.2)**	2013–2017	2.5 (−1.9 to 7.2)
Distant	157,175 (5)	7.2 (7.1 to 7.2)	2.2 (1.7 to 2.7)**	2003–2010	−0.7 (−1.5 to 0.2)	2010–2017	5.1 (4.3 to 5.8)**	—	—
Unknown	212,358 (7)	9.6 (9.6 to 9.7)	−2.8 (−5.3 to −0.2)**	2003–2005	−16.5 (−26.3 to −5.4)**	2005–2015	−3.8 (−5.4 to −2.1)**	2015–2017	19.1 (1.2 to 40.1)**
**Stage by race/ethnicity**									
**Localized**									
White	1,782,452 (78)	94.5 (94.3 to 94.6)	−3.4 (−5.3 to −1.4)**	2003–2007	3.1 (−1.3 to 7.8)	2007–2014	−8.4 (−10.7 to −6.1)**	2014–2017	0.5 (−7.1 to 8.7)
Black	349,321 (77)	153.8 (153.3 to 154.3)	−2.9 (−4.4 to −1.5)**	2003–2008	1.5 (−1.0 to 4.0)	2008–2014	−7.0 (−9.2 to −4.8)**	2014–2017	−1.7 (−7.0 to 3.9)
AI/AN	8,818 (72)	61.8 (60.4 to 63.2)	−3.9 (−6.2– to −1.6)**	2003–2008	1.1 (−3.0 to 5.3)	2008–2014	−9.0 (−12.5 to −5.3)**	2014–2017	−1.6 (−9.8 to 7.4)
API	45,682 (73)	48.9 (48.5 to 49.4)	−4.7 (−7.5 to −1.9)**	2003–2007	−0.4 (−7.4 to 7.1)	2007–2014	−8.9 (−12.2 to −5.5)**	2014–2017	−0.2 (−10.2 to 10.9)
Hispanic	143,627 (73)	76.3 (75.8 to 76.7)	−4.7 (−5.9 to −3.6)**	2003–2008	0.0 (−2.0 to 2.1)	2008–2014	−8.8 (−10.6 to −7.1)**	2014–2017	−4.1 (−8.4 to 0.4)
**Regional**									
White	267,155 (12)	13.5 (13.5 to 13.6)	0.5 (−1.5 to 2.5)	2003–2007	4.1 (−0.6 to 9.0)	2007–2013	−3.3 (−6.5 to −0.1)**	2013–2017	2.8 (−2.1 to 7.9)
Black	43,672 (10)	17.5 (17.3 to 17.6)	−0.1 (−2.1 to 2.0)	2003–2010	0.1 (−1.6 to 1.8)	2010–2013	−4.7 (−14.0 to 5.5)	2013–2017	3.3 (0.0 to 6.6)**
AI/AN	1,412 (12)	8.7 (8.2 to 9.2)	−0.7 (−2.1 to 0.7)						
API	8,014 (13)	7.8 (7.6 to 8.0)	0.9 (−2.2 to 4.0)	2003–2011	1.1 (−1.0 to 3.1)	2011–2014	−8.1 (−20.8 to 6.7)	2014–2017	10.1 (2.8 to 17.9)**
Hispanic	21,853 (11)	10.3 (10.2 to 10.5)	−1.4 (−2.0 to −0.9)**	2003–2017	−1.4 (−2.0 to −0.9)**	—		—	
**Distant**									
White	110,453 (5)	6.4 (6.3 to 6.4)	2.7 (2.1 to 3.2)**	2003–2010	−0.2 (−1.1 to 0.8)	2010–2017	5.6 (4.8 to 6.4)**	—	—
Black	28,946 (6)	15.1 (14.9 to 15.2)	0.1 (−0.6 to 0.8)	2003–2011	−2.4 (−3.4 to −1.3)**	2011–2017	3.5 (2.2 to 4.8)**	—	—
AI/AN	911 (7)	7.7 (7.1 to 8.2)	2.2 (0.8 to 3.6)**						—
API	3,867 (6)	4.7 (4.6 to 4.9)	1.7 (−0.5 to 4.0)	2003–2006	3.7 (−4.4 to 12.5)	2006–2010	−5.0 (−10.8 to 1.1)	2010–2017	4.9 (3.3 to 6.6)**
Hispanic	12,275 (6)	7.5 (7.4 to 7.6)	0.5 (−0.3 to 1.3)	2003–2011	−1.6 (−2.9 to −0.4)**	2011–2017	3.4 (2.0 to 4.8)**	—	
**Unknown**									
White	136,745 (6)	7.8 (7.8 to 7.9)	−6.1 (−9.0 to −3.1)**	2003–2005	−19.2 (−35.1 to 0.7)	2005–2017	−3.8 (−5.7 to −1.8)**	—	—
Black	29,883 (7)	16.0 (15.8 to 16.2)	−4.2 (−5.7 to −2.7)**	—	—	—	—	—	—
AI/AN	1,091 (9)	9.8 (9.2 to 10.4)	−6.0 (−8.4 to −3.5)**	—	—	—	—	—	—
API	4,621 (7)	5.7 (5.5 to 5.8)	−2.0 (−3.7 to −0.2)**	—	—	—	—	—	—
Hispanic	18,751 (10)	11.9 (11.7 to 12.1)	−4.2 (−6.1 to −2.2)**	—	—	—	—	—	—

**Abbreviations:** AI/AN = American Indian/Alaska Native; API = Asian/Pacific Islander; CI = confidence interval.

* Incidence data are compiled from cancer registries that meet the U.S. Cancer Statistics publication criteria for the period 2003–2017 (covering 100% of the U.S. population). Characteristic values with other, missing, or blank results are not included. Rates are age-adjusted to the 2000 U.S. Standard population.

^†^ Cases included *International Classification of Diseases for Oncology, Third Edition* malignant cancers only.

^§^ Trends were considered to increase or decrease if p<0.05; otherwise trends were considered stable.

^¶^ Denominator for this column is 3,087,800, except for stage by race/ethnicity, where the denominator is the total number of cases for the respective race/ethnicity grouping.

** p<0.05.

^††^ Trend described for the period 2003–2017 by previous APC columns.

^§§^ White, Black, AI/AN, and API men are non-Hispanic. Hispanic men might be of any race. Counts exclude unspecified or unknown race/ethnicity. Excludes 67,696 cases with non-Hispanic unknown race.

^¶¶^ Defined by merged Summary Stage. https://www.cdc.gov/cancer/uscs/public-use/dictionary/merged-summary-stage.htm.

**TABLE 2:** Relative survival of men with prostate cancer, 1, 5, and 10 years after diagnosis — United States, 2001–2016*

Characteristic	No.	1-year relative survival % (95% CI)	5-year relative survival % (95% CI)	10-year relative survival % (95% CI)
**Overall**	**3,104,380**	**99.0 (98.9–99.0)**	**97.6 (97.5–97.6)**	**97.2 (97.2–97.3)**
**Age group (yrs)**				
≤49	83,692	99.3 (99.2–99.3)	96.7 (96.6–96.7)	95.6 (95.6–95.9)
50–54	214,757	99.6 (99.5–99.6)	97.8 (97.6–97.8)	96.9 (96.9–97.1)
55–59	407,302	99.7 (99.6–99.7)	98.4 (98.3–98.4)	98.0 (98.0–98.1)
60–64	559,872	99.7 (99.7–99.7)	98.8 (98.8–98.8)	98.7 (98.7–98.9)
65–69	650,004	99.9 (99.9–99.9)	99.6 (99.5–99.6)	99.5 (99.5–99.7)
70–74	525,876	99.8 (99.8–99.8)	99.5 (99.4–99.5)	99.4 (99.4–99.6)
75–79	361,735	99.1 (99.0–99.1)	98.4 (98.2–98.4)	97.9 (97.9–98.3)
≥80	301,315	92.1 (92.0–92.1)	84.6 (84.2–84.6)	82.7 (82.7–83.5)
**Race/Ethnicity^†^**				
White	2,323,828	99.1 (99.0–99.1)	97.9 (97.9–97.9)	97.8 (97.8–97.9)
Black	459,665	98.4 (98.4–98.4)	95.6 (95.4–95.6)	93.5 (93.5–93.8)
AI/AN	11,983	98.2 (97.7–98.2)	95.7 (94.7–95.7)	93.4 (93.4–95.0)
API	55,310	98.7 (98.6–98.7)	95.1 (94.8–95.1)	92.0 (92.0–92.6)
Hispanic	193,770	98.4 (98.3–98.4)	95.3 (95.1–95.3)	93.1 (93.1–93.4)
**Stage^§^**				
Localized	2,393,365	100.0^¶^	100.0	100.0
Regional	328,421	100.0 (100.0–100.0)	98.6 (98.5–98.6)	96.1 (96.1–96.4)
Distant	145,923	75.6 (75.3–75.6)	30.7 (30.4–30.7)	18.5 (18.5–18.9)
Unknown	236,919	93.2 (93.1–93.2)	84.3 (84.0–84.3)	78.1 (78.1–78.5)
**Stage by age group (yrs)**				
**Localized**				
≤49	65,134	100.0 (99.8–100.0)	99.9 (99.7–99.9)	99.8 (99.8–99.9)
50–54	167,635	100.0	100.0	100.0
55–59	318,323	100.0	100.0	100.0
60–64	437,309	100.0	100.0	100.0
65–69	512,706	100.0	100.0	100.0
70–74	421,401	100.0	100.0	100.0
75–79	283,797	100.0	100.0	100.0
≥80	187,081	100.0 (99.9–100.0)	100.0 (99.9–100.0)	100.0 (100.0–100.0)
**Regional**				
≤49	12,140	99.8 (99.6–99.8)	97.0 (96.5–97.0)	92.6 (92.6–93.4)
50–54	32,016	100.0	97.9 (97.6–97.9)	94.1 (94.1–94.6)
55–59	58,398	100.0	99.0 (98.7–99.0)	95.9 (95.9–96.4)
60–64	76,162	100.0	100.0 (91.2–100.0)	97.8 (97.8–98.2)
65–69	77,433	100.0	100.0	99.9 (99.9–100.0)
70–74	42,562	100.0	100.0	99.6 (99.6–100.0)
75–79	17,034	99.3 (98.8–99.3)	94.2 (93.1–94.2)	90.4 (90.4–92.3)
≥80	12,678	90.7 (89.9–90.7)	70.8 (69.0–70.8)	64.4 (64.4–67.3)
**Distant**				
≤49	3,083	84.9 (83.6–84.9)	31.1 (29.2–31.1)	19.0 (19.0–20.9)
50–54	6,488	85.4 (84.5–85.4)	32.7 (31.3–32.7)	19.1 (19.1–20.5)
55–59	12,607	84.3 (83.6–84.3)	35.5 (34.5–35.5)	20.8 (20.8–21.9)
60–64	18,268	83.2 (82.6–83.2)	35.1 (34.2–35.1)	21.8 (21.8–22.8)
65–69	21,311	82.4 (81.8–82.4)	36.2 (35.3–36.2)	21.2 (21.2–22.1)
70–74	21,066	77.9 (77.3–77.9)	33.3 (32.5–33.3)	19.8 (19.8–20.9)
75–79	21,299	73.4 (72.8–73.4)	29.9 (29.0–29.9)	18.5 (18.5–19.6)
≥80	41,810	63.3 (62.8–63.3)	22.5 (21.8–22.5)	14.6 (14.6–15.8)
**Unknown**				
≤49	3,340	97.8 (97.2–97.8)	91.7 (90.5–91.7)	88.3 (88.3–89.8)
50–54	8,625	98.3 (98.0–98.3)	93.0 (92.2–93.0)	88.7 (88.7–89.8)
55–59	17,984	98.2 (97.9–98.2)	93.0 (92.4–93.0)	88.7 (88.7–89.5)
60–64	28,148	97.8 (97.5–97.8)	92.1 (91.6–92.1)	87.5 (87.5–88.3)
65–69	38,573	97.5 (97.3–97.5)	91.3 (90.8–91.3)	85.4 (85.4–86.2)
70–74	40,864	96.6 (96.3–96.6)	89.2 (88.6–89.2)	82.8 (82.8–83.8)
75–79	39,622	94.2 (93.9–94.2)	85.6 (84.9–85.6)	77.7 (77.7–79.0)
≥80	59,766	82.7 (82.3–82.7)	65.7 (64.9–65.7)	57.2 (57.2–58.7)

**Source:** CDC’s National Program of Cancer Registries, https://www.cdc.gov/cancer/npcr.

**Abbreviations:** AI/AN = American Indian/Alaska Native; API = Asian/Pacific Islander; CI = confidence interval.

* Data were compiled from 45 population-based registries that cover approximately 94% of the US population. Counts for age and stage do not sum to the total because of multiple primaries methodology. When the relative survival is calculated stratified by a tumor or demographic characteristic, each cancer was included for patients diagnosed with multiple primary prostate cancers at the different category-levels.

^†^ White, Black, AI/AN, and API men are non-Hispanic. Hispanic men might be of any race. Counts exclude unspecified or unknown race/ethnicity. Excludes 59,824 cases of non-Hispanic unknown race.

^§^ Percentage of total for localized, regional, distant, and unknown is 77%, 11%, 5%, and 8%, respectively.

^¶^ CI could not be calculated.

**TABLE 3:** Five-year relative survival for men with prostate cancer, by period and selected characteristics — United States, 2001–2016*

Characteristic	2001–2016		2001–2005		2006–2010		2011–2016	
	No.	Relative survival % (95% CI)	No.	Relative survival % (95% CI)	No.	Relative survival % (95% CI)	No.	Relative survival % (95% CI)
**Overall**	**3,104,380**	**97.6 (97.5–97.6)**	**965,748**	**97.3 (97.2–97.4)**	**1,052,255**	**98.2 (98.1–98.3)**	**1,086,532**	**97.2 (97.1–97.3)**
**Age group (yrs)**								
≤49	83,692	96.7 (96.6–96.9)	25,688	96.3 (96.0–96.6)	31,384	97.1 (96.8–97.3)	26,621	96.9 (96.5–97.2)
50–54	214,757	97.8 (97.6–97.9)	63,318	97.8 (97.6–98.0)	76,549	97.9 (97.7–98.1)	74,893	97.4 (97.2–97.7)
55–59	407,302	98.4 (98.3–98.5)	117,213	98.6 (98.4–98.7)	143,170	98.6 (98.5–98.7)	146,920	97.7 (97.5–97.9)
60–64	559,872	98.8 (98.8–98.9)	154,088	98.7 (98.6–98.9)	195,058	99.2 (99.1–99.4)	210,727	98.4 (98.2–98.6)
65–69	650,004	99.6 (99.5–99.7)	185,518	99.1 (98.9–99.3)	213,975	99.9 (99.7–100.0)	250,514	99.5 (99.3–99.6)
70–74	525,876	99.5 (99.4–99.6)	175,220	99.1 (98.8–99.3)	171,457	99.9 (99.9–100.0)	179,201	99.3 (99.0–99.4)
75–79	361,735	98.4 (98.2–98.6)	134,039	98.0 (97.6–98.3)	120,166	99.0 (98.7–99.2)	107,532	97.7 (97.4–98.0)
≥80	301,315	84.6 (84.2–84.9)	110,671	85.7 (85.2–86.3)	100,506	86.9 (86.3–87.4)	90,144	79.7 (78.9–80.6)
**Race/Ethnicity^†^**								
White	2,323,828	97.9 (97.9–98.0)	752,786	97.8 (97.7–97.9)	792,482	98.6 (98.5–98.6)	778,682	97.3 (97.2–97.5)
Black	459,665	95.6 (95.4–95.7)	130,818	94.8 (94.6–95.1)	152,416	96.1 (95.9–96.3)	176,445	95.7 (95.3–95.9)
AI/AN	11,983	95.7 (94.7–96.6)	3,361	94.6 (92.7–96.1)	3,991	96.4 (94.8–97.5)	4,632	95.5 (93.2–97.1)
API	55,310	95.1 (94.8–95.5)	14,865	95.4 (94.7–96.0)	18,207	95.5 (94.9–96.0)	22,241	94.7 (93.9–95.3)
Hispanic	193,770	95.3 (95.1–95.5)	52,951	94.9 (94.5–95.2)	64,680	96.0 (95.7–96.3)	76,154	95.2 (94.8–95.6)
Stage*								
Localized	2,393,365	100.0^§^	753,909	100.0^§^	836,008	100.0^§^	803,466	100.0^§^
Regional	328,421	98.6 (98.5–98.7)	87,320	97.5 (97.3–97.8)	106,635	99.0 (98.8–99.2)	134,467	99.3 (98.9–99.5)^¶^
Distant	145,923	30.7 (30.4–31.0)	37,195	28.7 (28.1–29.2)	40,895	30.2 (29.7–30.8)	67,835	32.3 (31.6–33.0)^¶^
Unknown	236,919	84.3 (84.0–84.5)	87,357	83.0 (82.6–83.4)	68,748	84.2 (83.7–84.6)	80,818	86.7 (86.1–87.2)^¶^
**Stage by race/ethnicity**								
**Localized **								
White	1,807,824	100.0^§^	592,631	100.0^§^	634,465	100.0^§^	580,741	100.0^§^
Black	354,643	100.0^§^	99,964	100.0^§^	121,260	100.0^§^	133,420	100.0^§^
AI/AN	8,626	100.0^§^	2,477	99.9 (97.3–100.0)	2,960	100.0^§^	3,189	100.0^§^
API	41,192	99.6 (99.1–99.8)	11,622	99.6 (98.1–99.9)	13,940	99.6 (98.6–99.9)	15,631	99.8 (97.4–100.0)
Hispanic	142,007	100.0^§^	39,557	100.0^§^	48,798	100.0^§^	53,655	100.0^§^
**Regional**								
White	254,394	98.6 (98.5–98.8)	68,723	97.5 (97.2–97.7)	83,382	99.0 (98.7–99.2)	102,290	99.4 (99.0–99.7)^¶^
Black	42,843	98.8 (98.3–99.1)	11,027	97.9 (96.9–98.5)	13,494	99.3 (98.3–99.7)	18,322	98.9 (97.7–99.5)
AI/AN	1,366	98.2 (93.2–99.5)	354	97.3 (87.8–99.4)	431	98.5 (82.2–99.9)	581	97.5 (79.3–99.7)
API	6,671	97.4 (96.5–98.1)	1,491	97.1 (95.0–98.4)	2,091	97.9 (96.3–98.9)	3,089	96.9 (94.7–98.2)
Hispanic	20,794	97.5 (97.0–98.0)	5,145	96.8 (95.7–97.7)	6,424	97.9 (96.9–98.5)	9,225	98.0 (96.7–98.8)
**Distant **								
White	101,621	29.1 (28.7–29.5)	25,864	27.2 (26.6–27.9)	28,392	28.5 (27.9–29.1)	47,367	30.8 (29.9–31.6)^¶^
Black	28,330	31.6 (30.9–32.3)	7,718	29.9 (28.6–31.1)	8,047	31.0 (29.9–32.2)	12,565	33.3 (31.8–34.9)^¶^
AI/AN	796	32.2 (27.8–36.8)	180	29.0 (21.6–36.8)	219	27.9 (21.3–34.8)	397	39.0 (30.4–47.4)
API	3,153	42.0 (39.6–44.3)	650	38.1 (33.9–42.3)	853	43.3 (39.5–47.0)	1,650	41.5 (36.8–46.1)
Hispanic	11,418	37.2 (36.1–38.4)	2,655	35.3 (33.3–37.4)	3,213	37.4 (35.5–39.3)	5,550	37.5 (35.2–39.9)
**Unknown**								
White	160,180	82.8 (82.5–83.2)	65,593	83.3 (82.8–83.8)	46,266	82.0 (81.4–82.5)	48,322	83.0 (82.2–83.8)
Black	33,879	79.1 (78.3–79.8)	12,113	78.3 (77.1–79.5)	9,619	78.8 (77.5–80.0)	12,148	80.9 (79.3–82.4)
AI/AN	1,196	82.2 (78.1–85.7)	350	76.9 (69.4–82.8)	381	84.6 (77.6–89.6)	465	84.1 (74.8–90.3)
API	4,298	82.7 (80.9–84.4)	1,102	82.0 (78.5–84.9)	1,323	81.1 (78.0–83.7)	1,873	85.4 (81.5–88.5)
Hispanic	19,572	84.4 (83.5–85.2)	5,598	80.6 (79.0–82.1)	6,249	86.5 (85.1–87.7)	7,727	85.8 (83.9–87.6)^¶^

**Source:** CDC’s National Program of Cancer Registries. https://www.cdc.gov/cancer/npcr.

**Abbreviations:** AI/AN = American Indian/Alaska Native; API = Asian/Pacific Islander; CI = confidence interval.

* Data were compiled from 45 population-based registries that cover approximately 94% of the U.S. population. Counts for age and stage do not sum to the total because of multiple primaries methodology. When the relative survival is calculated stratified by a tumor or demographic characteristic, each cancer was included for patients diagnosed with multiple primary prostate cancers at the different category levels.

^†^White, Black, AI/AN, and API men are non-Hispanic. Hispanic men might be of any race. Counts exclude unspecified or unknown race/ethnicity. Excludes 59,824 cases of non-Hispanic unknown race.

^§^ CI could not be calculated.

^¶^ Indicates nonoverlapping 95% CIs when comparing 2001–2005 with 2011–2016.

## Footnotes

* https://www.cdc.gov/cancer/uscs.

† https://www.cdc.gov/cancer/uscs/public-use/dictionary/behavior-code-ICD-O-3.htm.

§ https://seer.cancer.gov/siterecode/icdo3_dwhoheme/index.html.

¶ Rates were adjusted to the 2000 U.S. standard population.

** A maximum of two joinpoints were used to determine a change in direction of trend.

†† Registries met USCS publication criteria and included all U.S. states and the District of Columbia except for Connecticut, Hawaii, Indiana, Iowa, Kansas, and New Mexico.

§§ The cohort method was used to estimate survival when all patients had a full 1, 5, and 10 years of follow-up. The complete method was used when not all patients had the full 5 or 10 years of follow-up for 5-year and 10-year survival time estimates. https://surveillance.cancer.gov/survival/cohort.html.

¶¶ https://www.seer.cancer.gov.

*** Defined by merged Summary Stage. https://www.cdc.gov/cancer/uscs/public-use/dictionary/merged-summary-stage.htm and https://training.seer.cancer.gov/collaborative/intro/systems_review.html.

††† Information about the benefits and harms of prostate cancer screening can be found at the CDC website. Digital rectal examination to screen for prostate cancer is not recommended by USPSTF because of lack of evidence of the benefits. https://www.cdc.gov/cancer/prostate/basic_info/benefits-harms.htm, https://www.uspreventiveservicestaskforce.org/uspstf/recommendation/prostate-cancer-screening-2012, https://www.uspreventiveservicestaskforce.org/uspstf/recommendation/prostate-cancer-screening.

§§§ https://link.springer.com/article/10.1023/A:1023002322935.

¶¶¶ https://seer.cancer.gov/tools/collabstaging/.

**** https://www.uspreventiveservicestaskforce.org/uspstf/recommendation/prostate-cancer-screening.
